# Supplementary figures and images for: Transcriptome analysis of transcription factors and enzymes involved in monoterpenoid biosynthesis in different chemotypes of Mentha haplocalyx Briq
Source: PeerJ. 2023 Feb 20;11:e14914. doi: 10.7717/peerj.14914 (PMC9948755; doi:10.7717/peerj.14914)

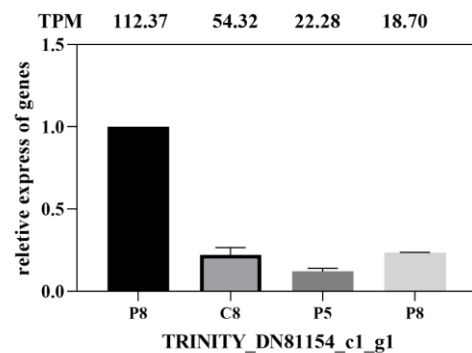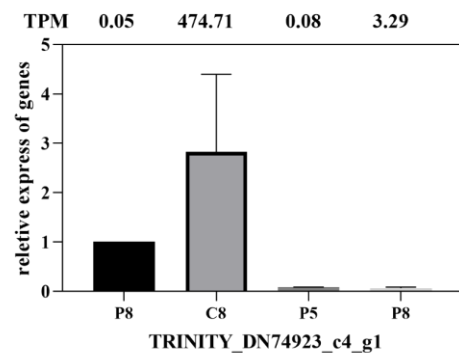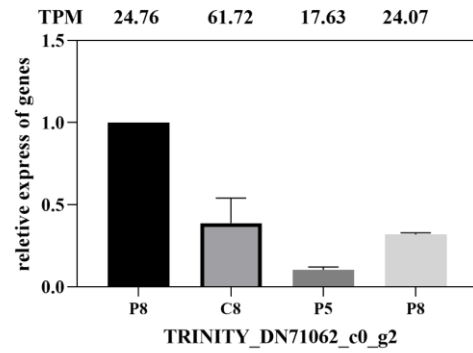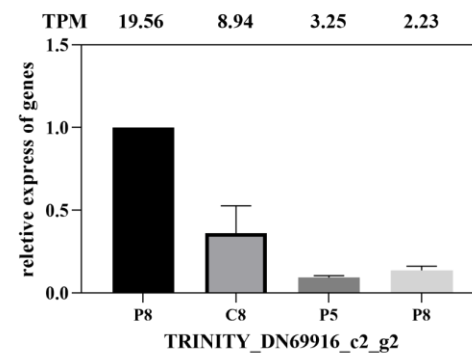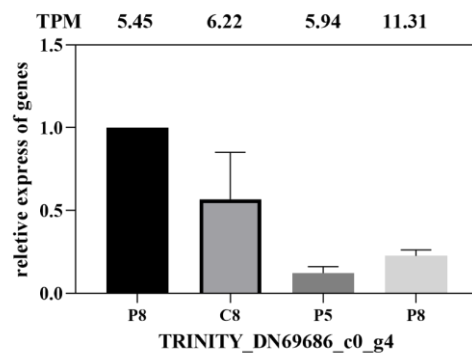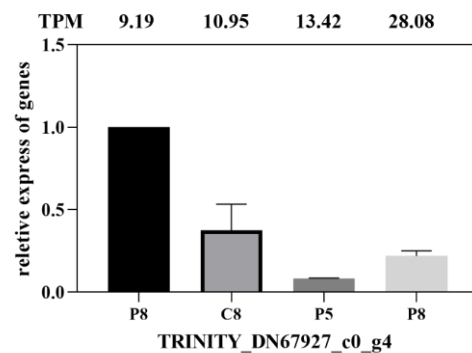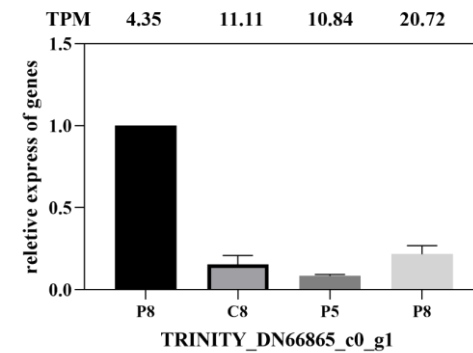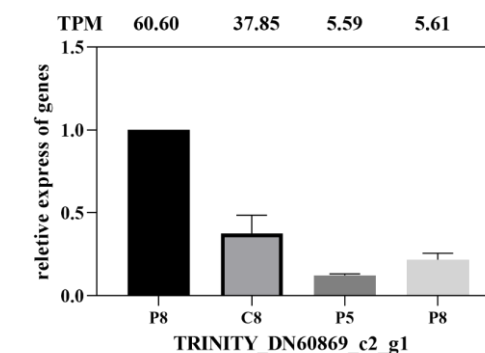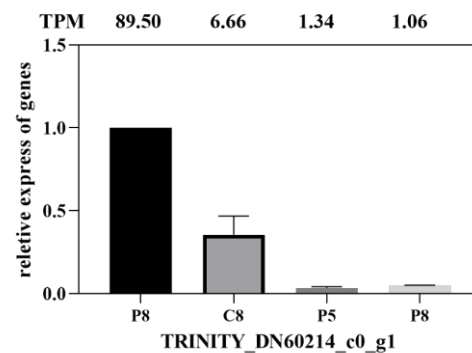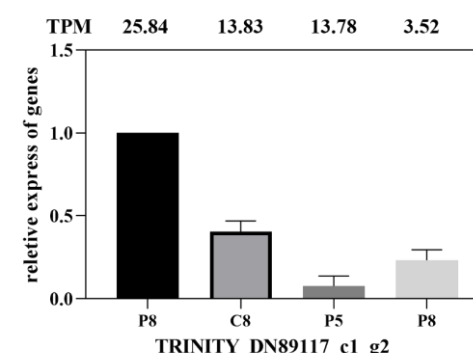

Supplement: Supplemental Information 2 [file peerj-11-14914-s002.pdf]

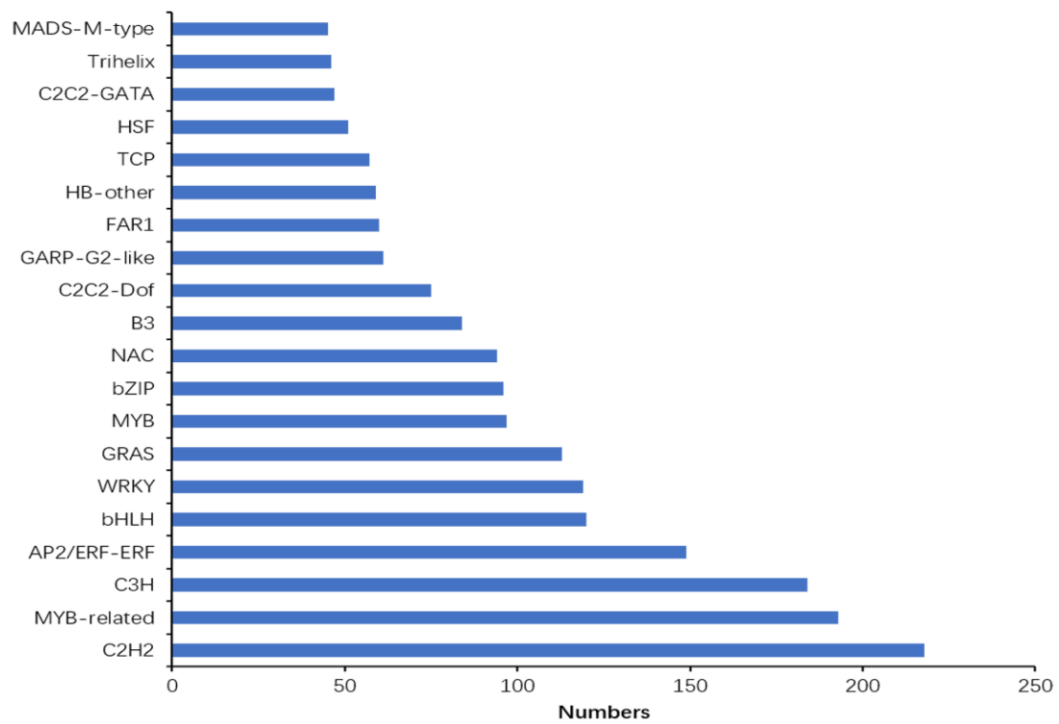

Supplement: Supplemental Information 3 — (A) M8 vs P8. (B) M8 vs C8. (C) M8 vs P5. [file peerj-11-14914-s003.pdf]

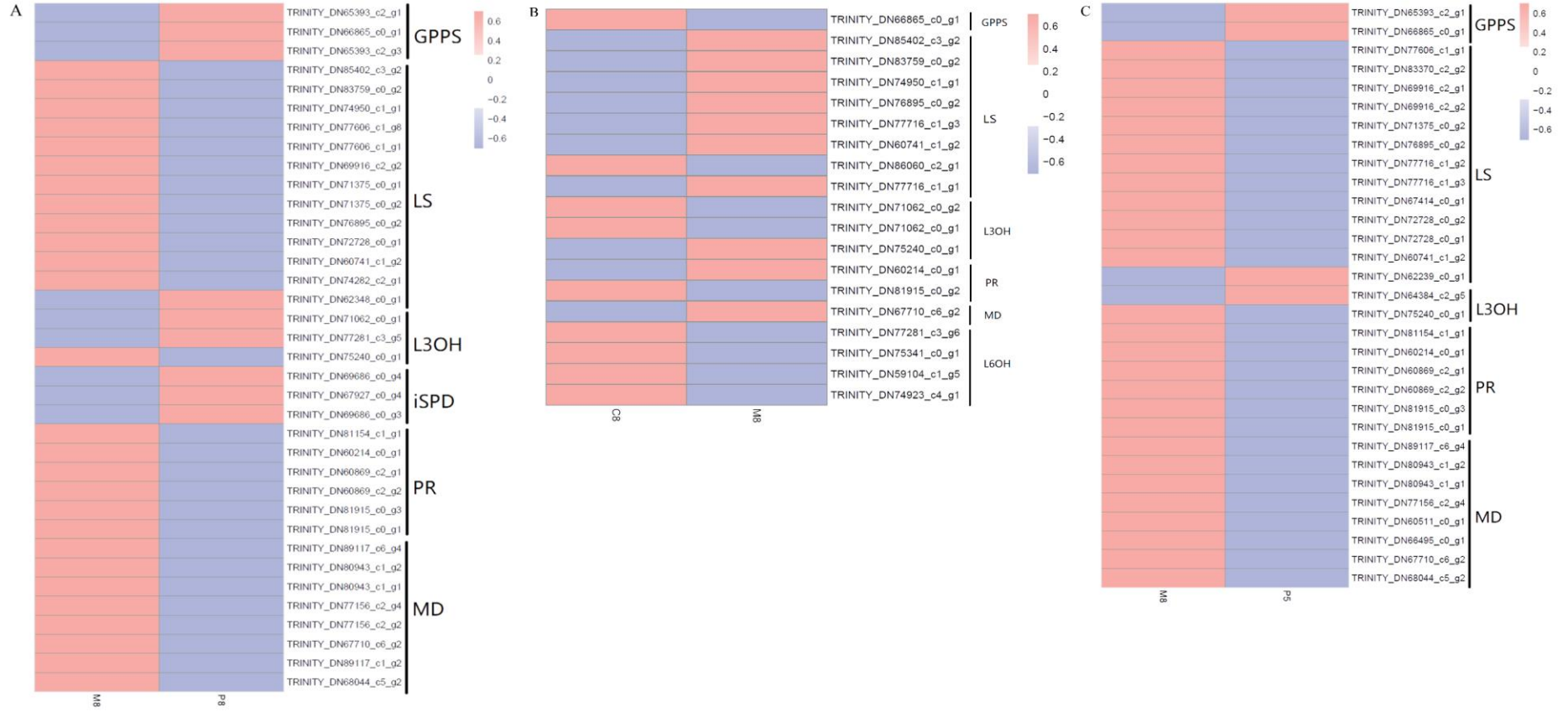

Supplement: Supplemental Information 4 [file peerj-11-14914-s004.pdf]
