# Supplementary material for: Transcriptome analysis of transcription factors and enzymes involved in monoterpenoid biosynthesis in different chemotypes of Mentha haplocalyx Briq
Source: PeerJ. 2023 Feb 20;11:e14914. doi: 10.7717/peerj.14914 (PMC9948755; doi:10.7717/peerj.14914)
Supplement: Supplemental Information 5 [file peerj-11-14914-s005.docx]

Table S1. Information on collection dates and local climate temperatures.

| Collection date | Maximum temperature℃ | Minimum temperature℃ | average temperature℃ |
| --- | --- | --- | --- |
| 20200510 | 24 | 15 | 19.5 |
| 20200520 | 30 | 18 | 24.0 |
| 20200528 | 34 | 22 | 28.0 |
| 20200608 | 32 | 23 | 27.5 |
| 20200619 | 26 | 21 | 23.5 |
| 20200630 | 29 | 21 | 25 |
| 20200708 | 30 | 23 | 26.5 |
| 20200720 | 29 | 22 | 25.5 |
| 20200731 | 31 | 27 | 29 |
| 20200805 | 34 | 27 | 30.5 |
| 20200812 | 35 | 27 | 31 |
| 20200821 | 33 | 26 | 29.5 |
| 20200903 | 30 | 20 | 25 |
| 20200914 | 26 | 21 | 23.5 |
| 20200928 | 25 | 19 | 22 |
| 20201013 | 23 | 15 | 19 |
| 20201023 | 18 | 7 | 12.5 |
| 20201031 | 21 | 13 | 17 |
