# Supplementary material for: Transcriptome analysis of transcription factors and enzymes involved in monoterpenoid biosynthesis in different chemotypes of Mentha haplocalyx Briq
Source: PeerJ. 2023 Feb 20;11:e14914. doi: 10.7717/peerj.14914 (PMC9948755; doi:10.7717/peerj.14914)
Supplement: Supplemental Information 6 [file peerj-11-14914-s006.docx]

Table S2 RIN value of RNA-seq data.

| Samples | Concentration(ng/μL) | RIN value |
| --- | --- | --- |
| M8-1 | 3267 | 7.4 |
| M8-2 | 1946 | 8.3 |
| M8-3 | 3126 | 8.3 |
| P8-1 | 650 | 9.1 |
| P8-2 | 1772 | 8.4 |
| P8-3 | 2735 | 8.4 |
| C8-1 | 789 | 7.4 |
| C8-2 | 2517 | 8.1 |
| C8-3 | 1548 | 8.4 |
| P5-1 | 2132 | 8.6 |
| P5-2 | 2667 | 7.6 |
| P5-3 | 1102 | 8.2 |
