# Supplementary material for: Transcriptome analysis of transcription factors and enzymes involved in monoterpenoid biosynthesis in different chemotypes of Mentha haplocalyx Briq
Source: PeerJ. 2023 Feb 20;11:e14914. doi: 10.7717/peerj.14914 (PMC9948755; doi:10.7717/peerj.14914)
Supplement: Supplemental Information 7 [file peerj-11-14914-s007.docx]

Table S3 Primers used in this investigation.

| Enzyme | Unigene ID | Forward primers（5’-3’） | Reverse primers（5’-3’） |
| --- | --- | --- | --- |
| GPPS | TRINITY_DN66865_c0_g1 | TCGAGTTCATCCACCACCAC | CCGTCTTCCCCAATTCCTTG |
|  | TRINITY_DN65393_c2_g3 | TTGTTGAACTTCCCGCACAG | CAACTATCACTGCAGGCGTC |
| LS | TRINITY_DN83370_c2_g2 | AGAGGCCCAACATGAATCCA | CATTCCACCAGTCTGTCCCT |
|  | TRINITY_DN69916_c2_g2 | GGTGAGTTCAAAGCAAGCCT | CAGCTCCACCTTCCTTCACT |
|  | TRINITY_DN67414_c0_g1 | CAAGAACGAGGAGGGTGAGT | AAAAGGTCGCCATCAACACC |
|  | TRINITY_DN72728_c0_g2 | CAAGAACGAGGAGGGTGAGT | CCACCACCCTCATTCACTCT |
|  | TRINITY_DN72728_c0_g1 | GCCGAAATCAATCCAGTGCT | GCGCCATTCTTCCCAGATTC |
| L3OH | TRINITY_DN71062_c0_g2 | ACGTCTGGTCTATGGGAAGG | CAATGGAACCTCGACGTTGG |
| PR | TRINITY_DN60214_c0_g1 | TGTCGTTGAGAACTTCCACG | AACACTCACAGATCCAGGAAG |
|  | TRINITY_DN81154_c1_g1 | GATTCGTGTGCAGGGGTTTC | TTCTTTCCCACGTTGTCACC |
|  | TRINITY_DN60869_c2_g1 | CGAAGCCGCGATCAATAATATG | TCAACAACGACGAAGCCTTG |
|  | TRINITY_DN60869_c2_g2 | TTGGGTTCGACGATGCATTT | TTTTCACGCCTTCAAGCTCC |
|  | TRINITY_DN81915_c0_g3 | ACAAGCTGGATGGGAGGAAT | TGCAAACTGACCAACAAGCT |
| MD | TRINITY_DN67710_c6_g2 | TCGTGCAAAACTTCCTCCAC | AGCCCTAATTCCTCTCCTGC |
|  | TRINITY_DN89117_c1_g2 | CTCGGATCATAGCGCGAAAG | GAGAGAAGAAGGAGCCCGAG |
| NMD | TRINITY_DN89117_c6_g4 | GCTGATCCTGCTAACCTTGC | GTACTCCACCATGACCAGCT |
|  | TRINITY_DN80943_c1_g1 | ACTGGTGCGAACAAAGGAAT | GCAAGGCTAGCAGGATCAGT |
| CD | TRINITY_DN67927_c0_g4 | CGGGATTAGGGTTAACAGCG | TCATCCGAAGCCAGAAATGC |
| L6OH | TRINITY_DN74923_c4_g1 | GCGGATCTCTACCCTTCCTC | TTTTGATGTCGCTGCCCTTC |
| iSPD | TRINITY_DN69686_c0_g4 | TTAGGGTAAACTGCGTGTCG | CACCACCAAATCATGCCCC |
| ERF | TRINITY_DN78234_c2g1 | TTGTTGAACTTCCCGCACAG | CAACTATCACTGCAGGCGTC |
|  | TRINITY_DN80375_c2g2 | CGCCGTGATCAATTTCCCTG | TCCCCGACATCCCAATAACC |
|  | TRINITY_DN68362_c0g2 | GAAATCCGCCACCCTCTACT | TCCCTAGACACAAACCAGCT |
|  | TRINITY_DN78537_c3g1 | GGCAGCCCAGATGATTTTCC | CGGCAGCATCATCATCTCAT |
| AP2 | TRINITY_DN86922_c2g1 | TGGTTAGGGCGAAGTACTGG | TCCCATCTGCCAGTTCTACG |
| bHLH | TRINITY_DN70782_c2g1 | TCGATTGCCTCAGAGTTCGT | CCTCTCAGACAAGACGTGGT |
|  | TRINITY_DN81055_c2g2 | GGTCAAAACACTCTGTCACGG | GTGGGCTCCGAATTCCAAG |
|  | TRINITY_DN85127_c2g1 | CCAATGCGAGTAAGACCGAC | TGGAGCTGTTGTTGCATTCC |
| bZIP | TRINITY_DN84983_c2g1 | TCAAGAACAGGGAGAGTGCC | GAGCCAGTTTTGTGCAGGAT |
| MYB | TRINITY_DN81229_c2g1 | GGGAATGGCACCTCATCTCC | CTTCTTCCACTTGTTGCCGT |
|  | TRINITY_DN80889_c0g2 | GAGGATCGGAAAATCGCAGC | TGTTCGACCCGGTAACCTAG |
| MYB_related | TRINITY_DN72105_c0g1 | AGTGAAGAGAGAGAGAGCGG | ACTTGTCATCCTCGTCGTCC |
|  | TRINITY_DN58126_c1g1 | CAGCGTGGCAAAACAAGGT | GGTTGATGTCTTCCACCAGC |
|  | TRINITY_DN78913_c0g1 | TGGACAGCAGAAGAAGAAGC | GGGATCCAAGACCATTTGCC |
|  | TRINITY_DN79026_c1g2 | CCCGAGGAAGATGGACTAAAC | CGGTTAGTCAAGGCTTCACG |
|  | TRINITY_DN83505_c1g2 | GAAACGAGCGTGTACTGGAC | TTCAGGGGAGGCTTTTGAGT |
| WRKY | TRINITY_DN66800_c2g2 | TCGCAGAATAACCGAAGCTC | TATCGACATGGCTCCTCGTT |
|  | TRINITY_DN82430_c2g1 | CCTCGTCTGACCCTCAAACT | TGTAATGAAGGTGCTCGGGT |
| *β*-actin | - | GGATATTCAGCCCCTCGTCT | TCTCTTGGATTGAGCCTCGT |
