# Supplementary material for: Transcriptome analysis of transcription factors and enzymes involved in monoterpenoid biosynthesis in different chemotypes of Mentha haplocalyx Briq
Source: PeerJ. 2023 Feb 20;11:e14914. doi: 10.7717/peerj.14914 (PMC9948755; doi:10.7717/peerj.14914)
Supplement: Supplemental Information 8 [file peerj-11-14914-s008.docx]

Table S4 Assembly results.

|  | No. | >=500bp | >=1000bp | N50 | N90 | Max Len | Min Len | Total Len | Average Len |
| --- | --- | --- | --- | --- | --- | --- | --- | --- | --- |
| Transcript | 706098 | 271886 | 80636 | 677 | 270 | 16483 | 201 | 389723934 | 551.94 |
| Unigene | 254942 | 78147 | 25124 | 621 | 242 | 16483 | 201 | 128420176 | 503.72 |
