# Supplementary material for: Transcriptome analysis of transcription factors and enzymes involved in monoterpenoid biosynthesis in different chemotypes of Mentha haplocalyx Briq
Source: PeerJ. 2023 Feb 20;11:e14914. doi: 10.7717/peerj.14914 (PMC9948755; doi:10.7717/peerj.14914)
Supplement: Supplemental Information 11 [file peerj-11-14914-s011.docx]

Table S7 Differentially expressed genes of key enzymes in major monoterpenoids biosynthesis pathway of *M. haplocaly*x.

| Gene | Enzyme | KO id (EC: NO) | No. All | No. Up | No. Down |
| --- | --- | --- | --- | --- | --- |
| GPPS | geranyl diphosphate synthase | K14066（EC 2.5.1.1） | 31 | 0 | 3 |
| LS | (4S)-limonene synthase | K15088（EC 4.2.3.16） | 6 | 4 | 1 |
| L3OH | (-)-limonene 3-hydroxylase | K15089（EC 1.14.14.99） | 6 | 0 | 1 |
| iSPD | isopiperitenol dehydrogenase | K15090（EC 1.1  1.223） | 7 | 0 | 4 |
| iSPR | (-)-isopiperitenone reductase | K15091（EC 1.3.1.82） | 28 | 0 | 0 |
| MFS | (+)-menthofuran synthase | K15093（EC 1.14.14.143） | 4 | 0 | 0 |
| PR | (+)-pulegone reductase | K15092（EC 1.3.1.81） | 15 | 6 | 6 |
| MD | (-)-menthol dehydrogenase | K15094（EC 1.1.1.207） | 46 | 3 | 2 |
| NMD | (+)-neomenthol dehydrogenase | K15095（EC 1.1.1.208） | 35 | 5 | 3 |
| L6OH | (-)-limonene 6-hydroxylase | K14732（EC 1.14.14.51） | 8 | 1 | 3 |
| CD | carveol dehydrogenase | K14730（EC 1.1.1.243） | 8 | 0 | 0 |
